# Functional Outcome of Endovascular Treatment in Patients With Acute Ischemic Stroke With Large Vessel Occlusion: Mothership Versus Drip-and-Ship Model in a Portuguese Urban Region

**DOI:** 10.7759/cureus.32659

**Published:** 2022-12-18

**Authors:** Sérgio Ferreira Cristina, Alberto Fior, Marta Alves, Ana Luísa Papoila, Ana Paiva Nunes

**Affiliations:** 1 Internal Medicine, Hospital de Cascais Dr. José de Almeida, Lisbon, PRT; 2 Internal Medicine, Centro Hospitalar Universitário de Lisboa Central, Lisbon, PRT; 3 Epidemiology and Statistics Unit, Research Center, Centro Hospitalar Universitário de Lisboa Central, Lisbon, PRT

**Keywords:** disability, drip-and-ship, mothership, thrombectomy, stroke

## Abstract

Introduction

Endovascular treatment (EVT) with mechanical thrombectomy and acute carotid stenting has become an integral part of the treatment of acute ischemic stroke with large vessel occlusion. Despite being included in the most recent stroke guidelines, only comprehensive centers can offer EVT and thus patients frequently need to be transferred from primary hospitals. We aimed to assess which pre-hospital model of care - direct admission to a comprehensive stroke center (mothership) or transfer to a comprehensive stroke center after the first admission to the nearest hospital (drip-and-ship) - had the most benefit in stroke patients in a Portuguese urban region.

Methods

We selected patients admitted to a comprehensive stroke center who underwent EVTs between January 2018 and December 2020, in Lisbon, Portugal. We used data from the Safe Implementation of Treatments in Stroke (SITS) International registry on stroke severity, previous modified Rankin Scale (mRS), time from symptom onset to the first admission, time from symptom onset to the procedure, and mRS three months post stroke. We defined an unfavorable outcome as having an mRS >2 at three months post stroke. For patients with previous mRS >2, an unfavorable outcome was defined as any increase in mRS at three months post stroke.

Results

We analyzed the data of 1154 patients, of which 407 were admitted through a mothership approach and 747 through a drip-and-ship approach. Both groups were similar regarding sociodemographic characteristics, stroke risk factors, previous disability, and stroke severity. Median onset-to-door time was higher (126 vs 110 minutes, p-value=0.002) but onset-to-procedure time was lower (199 vs 339 minutes, p-value<0.001) in the mothership group. The mothership group had a higher proportion of patients with mRS <3 at three months post stroke than the drip-and-ship group (41.3% vs 34.9%, p-value=0.035). Mortality was similar in both groups. A multivariate logistic regression model confirmed a lower probability of unfavorable outcomes with the mothership approach (OR = 0.677, 95% CI 0.514-0.892, p-value=0.006). Surprisingly, onset-to-procedure time did not have an impact on functional outcomes.

Conclusion

Our findings show that the mothership model results in better functional outcomes for patients with acute ischemic stroke with large vessel occlusion. Further studies are needed to better define patient selection for this strategy and the impact of a mothership model in comprehensive stroke centers.

## Introduction

Acute stroke is a major health problem. In 2019, there were 12.22 million new cases of stroke, 101 million prevalent strokes, and 6.55 million deaths due to stroke [1]. Ischemic strokes are responsible for the majority (62.4%) of stroke episodes and achieving reperfusion with appropriate therapy is a fundamental step in the acute care of these patients [1-4].

For eligible patients, intravenous thrombolysis with alteplase (IV rt-PA) improves functional outcomes and should be administered in the first 4.5 hours of onset [2,3,5]. In the subset of patients with large vessel occlusion of the anterior circulation, the use of mechanical thrombectomy with second-generation stent retrievers and/or thrombus aspiration leads to improved functional outcomes and should be offered in addition to intravenous thrombolysis and in patients ineligible for thrombolysis [2,4,6]. Although the largest benefit is observed with mechanical thrombectomy in the first six hours of stroke onset, the DAWN and DEFUSE 3 trials also showed the benefit of mechanical thrombectomy in selected patients within the first 24 hours of stroke onset [7,8]. For patients with tandem lesions, acute carotid stenting also seems to improve functional outcomes [9,10].

There is the widespread use of alteplase in developed countries, but most hospitals do not have access to mechanical thrombectomy due to the need for experienced neurointerventionists and advanced equipment [11]. As such, stroke patients may need to be transported to hospitals capable of performing mechanical thrombectomy [2,4,11].

In thrombectomy-capable centers (mothership model), patients should be offered IV rt-PA without delaying mechanical thrombectomy [2,4]. In non-thrombectomy-capable centers, patients should receive IV rt-PA and be transferred to a thrombectomy-capable center (drip-and-ship model) [2,4]. Ideally, all patients with suspected stroke should be primarily transported to a thrombectomy-capable center but this strategy could delay the use of thrombolytic therapy if the thrombectomy-capable center is far from the location of the stroke onset, particularly during the one-hour frame when outcomes are better (golden hour) [12-14]. However, this strategy might have the advantage of a shorter time to reperfusion in patients submitted to mechanical thrombectomy, which is associated with better outcomes [15].

Several studies have compared outcomes from the use of the drip-and-ship and mothership models, and, although most seem to favor the mothership approach (particularly, due to higher functional independence), regional differences in geography and healthcare organization make it difficult to understand the benefit for each country and region [16-21].

We aimed to understand the differences in outcome with the drip-and-ship and mothership models in a Portuguese urban region.

## Materials and methods

Study design and participants selection

We conducted a retrospective observational study using data from the Safe Implementation of Treatments in Stroke (SITS) International registry, which is a prospective, multicenter, observational registry of treatments and outcomes of stroke patients. We selected patients with an ischemic stroke admitted to a comprehensive stroke center (CSC) between January 2018 and December 2020, in Lisbon, Portugal. Inclusion criteria were having a diagnosis of ischemic stroke with large vessel occlusion, receiving endovascular treatment (EVT) (mechanical thrombectomy and/or angioplasty and stent implantation) regardless of previous IV rt-PA, and being over 18 years old at the time of the stroke episode. We excluded patients transferred to other hospitals for EVT to reduce confounding factors due to different acute phase treatments in different stroke units.

Anonymity was assured for all participants. Our institutional ethics committee approved the study (Comissão de Ética para a Saúde approval number 1173/2021).

Data collection and statistical analysis

We collected data on the gender and age of the participants, the presence of risk factors for ischemic stroke, and previous disability using the modified Rankin Scale (mRS) for Neurological Disability [22]. We also collected data on the clinical characteristics of the stroke episode, particularly the National Institutes of Health Stroke Scale (NIHSS) at admission and 24 hours after admission, the type of EVT, the administration of IV rt-PA, the time from onset to hospital admission (“onset-to-door”), and the time from onset to EVT (“onset-to-procedure”) [23]. As for outcome measures, we collected data on the mRS three months after the stroke episode. Although a bad outcome has been frequently defined as having an mRS >2 at three months post stroke, since there are several patients with previous mRS >2 who undergo thrombectomy, we defined unfavorable outcomes differently according to the previous mRS. For patients with previous mRS <3, an unfavorable outcome was defined as having an mRS >2 at three months post stroke. For patients with previous mRS >2, an unfavorable outcome was defined as any increase in mRS at three months post stroke.

Participants were divided into two groups whether they were first admitted to the thrombectomy center (mothership group) or first admitted to a non-thrombectomy capable center with the following transfer to the thrombectomy center (drip-and-ship group). Data was compared using the Mann-Whitney U test, except for categorical variables which were compared with the χ2 test. All tests were two-tailed and statistical significance was set at p-value <0.05. For evaluating exposure effect on the outcome, we conducted univariate and multivariate analyses using logistic regression models. The CI was set at 95%. Statistical analysis was conducted using SPSS, version 22.0 (IBM Corp., Armonk, NY).

## Results

During the study period, there were 1217 patients admitted to the stroke unit after being submitted to EVT for ischemic stroke. Of these, 63 underwent EVT at another hospital and were thus excluded. Of the remainder 1154 patients, 407 were direct admissions to the CSC (mothership group) and 747 were transferred from other hospitals (drip- and-ship group).

Pre-procedure data of participants in both groups are represented in Table 1. Patients were slightly younger in the drip-and-ship group than in the mothership group (p-value=0.038). The median age was 78 years (P25=68, P75=85) in the mothership group and 76 years (P25=66, P75=83) in the drip-and-ship group. No difference was found between both groups in relation to gender (p-value=0.112). The mothership group had 56.0% of females and the drip-and-ship group had 51.1%. Both groups were also similar in previous disability (p-value=0.653). Most patients had an mRS of 0 in both the mothership group (76.4%) and the drip-and-ship group (77.0%). In the mothership group, 59 (14.5%) patients had an mRS >2 and in the drip-and-ship group 98 (13.1%) patients had an mRS >2.

**Table 1:** Pre-procedure characteristics of ischemic stroke patients with large vessel occlusion mRS: modified Rankin Scale for Neurological Disability, NIHSS: National Institutes of Health Stroke Scale

Pre-procedure characteristics		Mothership group (n=407)		Drip-and-ship group (n=747)		p-value
		n	%	n	%	
Gender	Female	228	56.0	382	51.1	0.112
	Male	179	44.0	365	48.9	
mRS before stroke	0	311	76.4	575	77.0	0.653
	1	17	4.2	47	6.3	
	2	20	4.9	27	3.6	
	3	33	8.1	65	8.7	
	4	24	5.9	33	4.4	
	5	2	0.5	0	0	
		n	Median (P_25_-P_75_)	n	Median (P_25_-P_75_)	
Age (years)		407	78 (68-85)	747	76 (66-83)	0.038
NIHSS at admission		406	15 (11-20)	724	16 (11-21)	0.389
NIHSS before procedure		399	15 (11-21)	715	16 (10-21)	0.854
Onset-to-Door time (minutes)		374	126 (84-315)	663	110 (68-231)	0.002
Onset-to-Procedure time (minutes)		364	199 (146-321)	675	339 (255-439)	<0.001

There was no difference in stroke severity between groups. Median NIHSS at admission was 15 (P25=11, P75=20) in the mothership group and 16 (P25=11, P75=21) in the drip-and-ship group (p-value=0.389). Similarly, median NIHSS before EVT was 15 (P25=11, P75=21) and 16 (P25=10, P75=21) in the mothership and drip-and-ship groups, respectively (p-value=0.854). Frequency of risk factors for ischemic stroke was similar between groups (Table 2). The exception was dyslipidemia, which seemed slightly higher in the drip-and-ship group (46.6% vs 40.5%, p-value=0.048). As expected, time from stroke onset to hospital admission (onset-to-door) was higher in the mothership group (median 126 minutes; P25=84, P75=314.5) than in the drip-and-ship group (median 110 minutes; P25=68, P75=231) (p-value=0.002), but the time from stroke onset to EVT (onset-to-procedure) was significantly lower in the mothership group (median 199 minutes; P25=146, P75=321) than in the drip-and-ship group (median 339 minutes; P25=255, P75=439) (p-value<0.001) (Table 2).

**Table 2:** Risk factors for ischemic stroke

Risk factors	Mothership group (n=407)		Drip-and-ship group (n=747)		p-value
	n	%	n	%	
Atrial fibrillation	189	46.7	349	46.7	0.986
Diabetes mellitus	84	20.7	188	25.2	0.087
Hypertension	311	76.4	564	75.5	0.730
Dyslipidemia	165	40.5	348	46.6	0.048
Heart failure	76	18.7	112	15.0	0.106
Smoking	36	8.9	71	9.5	0.725
Former smoking	21	5.3	46	6.4	0.466
Previous transient ischemic attack	6	1.5	14	1.9	0.621
Previous stroke within the latest 3 months	5	1.2	10	1.4	0.857
Previous stroke earlier than 3 months	54	13.3	100	13.4	0.955

Despite the significantly different time from stroke onset to hospital admission, treatment strategy was similar between both groups (p-value=0.265) (Table 3). In the mothership group, 183 patients were submitted to IV rt-PA and EVT (45.0%), 218 were submitted to EVT alone (53.6%), and six were treated with stent implantation (1.5%). In the drip-and-ship group, 372 patients were submitted to IV rt-PA and EVT (49.8%), 367 were submitted to EVT alone (49.1%), and eight were treated with stent implantation (1.1%).

**Table 3:** Treatment and outcome of ischemic stroke patients with large vessel occlusion EVT: endovascular treatment, IV rt-PA: intravenous thrombolysis with alteplase, mRS: modified Rankin Scale for Neurological Disability, NIHSS: National Institutes of Health Stroke Scale

Treatment and outcome variables			Mothership group (n=407)		Drip-and-ship group (n=747)		p-value
			n	%	n	%	
Treatment strategy	IV rt-PA + EVT		183	45.0	372	49.8	0.265
	EVT		218	53.6	367	49.1	
	Stent implantation		6	1.5	8	1.1	
mRS 3 months post-stroke		<3	161	41.3	244	34.9	0.035
		≥3	229	58.7	456	65.1	
Unfavorable outcome		No	183	46.9	265	37.9	0.004
		Yes	207	53.1	435	62.1	
3-month mortality			82	21.0	169	24.1	0.241
			n	Median (P_25_-P_75_)	n	Median (P_25_-P_75_)	
NIHSS 24 hours post-procedure			395	8 (2-17)	719	12 (4-19)	<0.001

Outcome analysis revealed better results in the mothership group (Table 3). Median NIHSS 24 hours after stroke onset was significantly lower in the mothership group (8 vs 12, p-value<0.001). Similarly, there was also a statistically significant difference in mRS score distribution three months after stroke onset (p-value=0.035), having a higher proportion of patients with minimal disability (mRS <3) three months after stroke onset in the mothership group (n=161, 41.3%) than in the drip-and-ship group (n=244, 34.9%). Even when considering previous disability, unfavorable outcome was also less frequent in the mothership group (n=207, 53.1%) than in the drip-and-ship group (n=435, 62.1%) (p-value=0.004). Three-month mortality was similar between groups (p-value=0.241).

We also analyzed how much the pre-hospital strategy impacted the probability of an unfavorable outcome (Table 4). Univariate analysis showed that patients in the mothership group had a lower probability of an unfavorable outcome than patients in the drip-and-ship group (OR=0.689, 95% CI 0.536-0.885, p-value=0.004). Risk factors associated with an unfavorable outcome were age above 80 (OR=2.501, 95% CI 1.932-3.239, p-value<0.001), atrial fibrillation (OR=1.756, 95% CI 1.374-2.244, p-value<0.001), hypertension (OR=1.936, 95% CI 1.460-2.568, p-value<0.001), and diabetes mellitus (OR=1.616, 95% CI 1.206-2.167, p-value=0.001). Current smokers seemed to have a lower probability of an unfavorable outcome in the univariate analysis (OR=0.532, 95% CI 0.353-0.800, p-value=0.002). As expected, more severe strokes (NIHSS>15 at hospital admission) were associated with a higher probability of an unfavorable outcome and stroke severity seemed to be the most important factor for the outcome (OR=3.847, 95% CI 2.973-4.978, p-value<0.001). Surprisingly, time from stroke onset to EVT did not seem to impact the probability of an unfavorable outcome (OR=1.000, 95% CI 1.000-1.001, p-value=0.377), even when dichotomized to those with more and less than 6 hours of stroke onset (OR=1.143, 95% CI 0.875-1.494, p-value=0.326).

**Table 4:** Univariate model of functional outcome at three months post stroke Note: continuous and ordinal variables are presented as median (P25-P75) and categorical variables as n (%). CI: confidence interval, mRS: modified Rankin Scale for Neurological Disability, NIHSS: National Institutes of Health Stroke Scale, TIA: transient ischemic attack

Variables	Favorable Outcome (reference)	Unfavorable Outcome	Odds Ratio	95% CI	p-value
Age (years)	73 (61-81)	79 (70-85)	1.039	1.029-1.049	<0.001
Age ≥80	124 (28.3%)	314 (71.7%)	2.501	1.932-3.239	<0.001
Female Gender	223 (38.7%)	353 (61.3%)	1.232	0.968-1.570	0.090
Atrial Fibrillation	173 (33.9%)	337 (66.1%)	1.756	1.374-2.244	<0.001
Hypertension	310 (37.3%)	522 (62.7%)	1.936	1.460-2.568	<0.001
Diabetes mellitus	85 (32.6%)	176 (67.4%)	1.616	1.206-2.167	0.001
Dyslipidemia	193 (39.5%)	296 (60.5%)	1.130	0.886-1.441	0.323
Current smoking	57 (55.3%)	46 (44.7%)	0.532	0.353-0.800	0.002
Previous smoking	31 (47.0%)	35 (53.0%)	0.779	0.473-1.284	0.327
Previous stroke earlier than 3 months	56 (38.6%)	89 (61.4%)	1.127	0.787-1.612	0.515
Previous stroke within the latest 3 months	8 (57.1%)	6 (42.9%)	0.520	0.179-1.510	0.229
Previous TIA	8 (42.1%)	11 (57.9%)	0.962	0.384-2.411	0.934
Heart failure	64 (36.2%)	113 (63.8%)	1.282	0.918-1.789	0.145
Previous mRS >2	54 (36.0%)	96 (64.0%)	1.283	0.897-1.835	0.172
Previous mRS	0 (0-0)	0 (0-1)	1.140	1.028-1.264	0.013
NIHSS at admission	12 (8-16)	18 (13-22)	1.137	1.113-1.163	<0.001
NIHSS at admission >15	145 (26.4%)	404 (73.6%)	3.847	2.973-4.978	<0.001
Onset-to-procedure time (minutes)	292.5 (197-403.5)	298.5 (215.25-415)	1.000	1.000-1.001	0.377
Onset-to-procedure time > 6 hours	135 (39.0%)	211 (61.0%)	1.143	0.875-1.494	0.326
Mothership model	183 (46.9%)	207 (53.1%)	0.689	0.536-0.885	0.004

Multivariate analysis confirmed the impact of the pre-hospital strategy on the outcome (Table 5). When adjusting for other variables, the mothership strategy still had a lower probability of an unfavorable outcome than the drip-and-ship strategy (OR=0.677, 95% CI 0.514-0.892, p-value=0.006). Only older age (OR=1.037, 95% CI 1.026-1.048, p-value<0.001), diabetes mellitus (OR=1.519, 95% CI 1.105-2.089, p-value=0.010), and NIHSS>15 at hospital admission (OR=3.626, 95% CI 2.778-4.732, p-value<0.001) were responsible for higher probability of an unfavorable outcome when adjusted for other variables.

**Table 5:** Multivariate model of functional outcome at three months post stroke Note: continuous and ordinal variables are presented as median (P25-P75) and categorical variables as n (%). CI: confidence interval, NIHSS: National Institutes of Health Stroke Scale

Variables	Favorable Outcome (reference)	Unfavorable Outcome	Odds Ratio	95% CI	p-value
Age	73 (61-81)	79 (70-85)	1.037	1.026-1.048	<0.001
Diabetes mellitus	85 (32.6%)	176 (67.4%)	1.519	1.105-2.089	0.010
NIHSS at admission >15	145 (26.4%)	404 (73.6%)	3.626	2.778-4.732	<0.001
Mothership model	183 (46.9%)	207 (53.1%)	0.677	0.514-0.892	0.006

## Discussion

There is still ongoing debate as to which model of pre-hospital care (mothership or drip-and-ship model) offers better outcomes and regional differences might play an important role in choosing either model [16-21].

As expected, our results show that, with either model, patient selection for EVT and chosen treatment is the same, but onset-to-door time is higher, and onset-to-procedure is lower with the mothership model. In this region, patients in the mothership group had better functional outcomes when compared to patients in the drip-and-ship group. Particularly, three months after the stroke episode, there was a higher proportion of patients with mRS <3 and a lower proportion of patients with an unfavorable outcome (irrespective of previous disability). The univariate logistic regression analyses also showed a lower probability of an unfavorable outcome with the mothership approach. Multivariate analysis confirmed the impact of pre-hospital strategy on the outcome, alongside stroke severity, patient age, and the presence of a previous diagnosis of diabetes mellitus.

Despite our original thoughts that the better results with the mothership groups were due to the lower time to EVT, onset-to-procedure time did not seem to be associated with functional outcomes in both the univariate and the multivariate logistic regression models. Although time from symptom onset is still considered an important factor when defining eligibility for EVT, infarct area, mismatch from clinical severity and infarct volume, the existence of effective collateral circulation, and other imaging and clinical criteria are also important factors for the successful outcome of EVT [7,8,24]. In our study, patient selection was performed by a stroke team and, although not evaluated in our study, the selection of patients with characteristics associated with benefit in EVT was probably more important than time criteria alone, particularly, since all patients were treated in the 24-hour recommended time window.

Our results also showed the negative impact most stroke risk factors have on outcome, although only advanced age and diabetes mellitus remained significant in the multivariate analysis. Surprisingly, smoking was associated with a lower probability of an unfavorable outcome, albeit this was not true in the multivariate analysis. Since our study was not specifically designed to evaluate risk factors' impact on the outcome, we cannot be certain whether this result represents a true protective effect. Additionally, there is published evidence suggesting the opposite effect. A study specifically aimed to evaluate the impact of smoking status on outcomes after acute ischemic stroke showed smoking was associated with worse outcomes [25].

There are several limitations to our study. All participants of our study were stroke patients with large vessel occlusion and, although we were able to ascertain that the mothership approach was beneficial for these patients, we were unable to state if this approach could be harmful due to delays in thrombolysis for patients not a candidate for EVT. Distance from the site of stroke onset to the EVT-capable center was also not assessed in our study and so we cannot determine if this approach is beneficial for all patients independent of distance to the hospital. However, we observed that patients in the mothership group (who were expected to be near the CSC) had higher onset-to-door times and yet better outcome results. In fact, it might be beneficial to have specific pre-hospital criteria to choose which patient benefits from early admission to a CSC regardless of the distance to it. In our country, pre-hospital teams use the Cincinnati Stroke Scale to identify potential strokes and bypass hospitals without a stroke fast-track, but there is no guidance on the identification of patients for EVT. The use of pre-hospital clinical tools to identify patients benefiting from EVT (e.g., ACT-FAST, RACE) might translate into a more accurate transfer to a CSC and improved reperfusion time and functional outcomes [26,27]. Additionally, in recent years, the use of mobile stroke units has been investigated as another pre-hospital strategy to improve outcomes. Mobile stroke units appear to improve outcomes in urban regions by improving time to thrombolysis [28,29]. Although still insufficient, there is already evidence that mobile stroke units might also benefit patients by earlier identification of EVT candidates and reduced onset-to-EVT times [30].

Another potential confounding factor is the presence of trained stroke teams at some but not all admission hospitals and thus acute stroke care might differ significantly between hospitals. In fact, in the mothership group, all patients were first approached by a trained stroke team at the emergency department and the better outcomes might, in part, be due to better acute care. Our study focused on pre-hospital strategy and as such, we did not assess imaging data (e.g., site of artery occlusion, Alberta Stroke Program Early CT Score [ASPECTS], perfusion studies), treatment complications (e.g., hemorrhagic transformation, systemic hemorrhage, puncture site complications), or recanalization status. These data might have had an impact on functional outcomes that were not assessed in the statistical models we used.

Our study lays the foundation for optimizing pre-hospital stroke care in Lisbon, Portugal, as no other published study, to our knowledge, addresses the benefit of the mothership strategy in this geographic area. It would be important to confirm these results with a randomized controlled trial comparing both strategies and further define what is the optimal distance for applying the mothership model and the burden this strategy could have on the emergency departments of CSC. The results of these studies should lead to a change in organizational policies to improve outcomes in acute stroke patients.

## Conclusions

Our study showed that, in the urban region of Lisbon, Portugal, a mothership model results in better functional outcomes for patients with acute ischemic stroke candidates for EVT. Further studies are needed to understand how to select patients for this strategy in the pre-hospital setting, specifically those who might have a large vessel occlusion and at what distance is the mothership approach most beneficial, and how an organizational change to a mothership model might impact the functioning of emergency departments and stroke units at comprehensive stroke centers.
